# Clinical and Patient Satisfaction Outcomes from Southern Osseointegrated Fixtures for Craniofacial Reconstruction: A Retrospective Study

**DOI:** 10.1097/SCS.0000000000013028

**Published:** 2026-06-18

**Authors:** Sara Moeini, Jack Brown, Morgan Marshall, Alexander Zargaran, Yazan Al-Ajam, Alex Woollard, Norbert Kang

**Affiliations:** * University College London; † Royal Free Hospital, London, UK

**Keywords:** Bone-anchored prosthetics, maxillofacial prosthesis implantation, osseointegration, prosthesis retention, reconstruction

## Abstract

This study reports the clinical outcomes from using Southern osseointegrated fixtures (SOFs) for retention of auricular, nasal, and orbital silicone prosthetics. Osseointegrated implants are widely used in the craniofacial skeleton, but data specific to the use of SOFs are more limited. A retrospective review was conducted of 16 patients who underwent implantation of SOFs between 2013 and 2024 at a large London teaching hospital. Data were collected from Electronic Patient Records and post-implantation surveys to assess patient satisfaction, prosthesis use, and complication rates. Descriptive statistical analysis was used to evaluate implant success, complications, and patient-reported outcomes (PROMS). A total of 58 implants were reviewed, with an average follow-up of 56 months. Implant retention was 82.76%; 10 implants failed to osseointegrate, predominantly in patients treated with radiotherapy to the underlying bone. The most common complications were over-granulation (8 implants), abutment or superstructure loosening (5 implants), and infection (4 implants). Of the 10 patients who completed a satisfaction survey, 8 were wearing at least one prosthesis daily, and all reported that their prosthetics were highly stable with an average satisfaction score of 4.88/5. Aesthetic outcomes were also high at 4.63/5. In this cohort, SOFs provided stable and effective fixation for craniofacial prosthetics, resulting in high patient satisfaction. Complications were generally infrequent and manageable, although implant failure in irradiated bone remains problematic.

Patients with acquired or congenital facial disfigurement often experience psychological distress.^1^ When reconstruction with autologous techniques is not possible,^2–4^ the best alternative is a prosthetic reconstruction.^5^ This aims to restore normal form but is seldom able to reproduce function.^6^

One of the main determinants of success with a prosthetic approach is the method used to hold the prosthesis in position. Past methods have included spectacles, silicone-based adhesives, or surgical interventions such as rotation flaps.^7,8^ However, the current state of the art is to use an osseointegrated (OI) implant, which permits direct skeletal fixation.^9,10^

Osseointegration is a well-understood biological phenomenon that has been studied for >60 years.^11^ Although other materials can achieve similar outcomes,^12^ the majority of implants that are commercially available are titanium-based. Importantly, the risks associated with the use of OI implants are small,^13,14^ and very small when compared with the risks associated with autologous reconstruction of similar defects.^15,16^ Mostly, the complications relate to infections of the soft tissues around the OI implants (rather than bony infection), localized pain, and implant loss.^17,18^ Even when they occur, the risks are infrequent and easy to deal with, especially when the appropriate postoperative protocols are followed.^19^

Although OI implants have been in use for many years, there are few publications related to the use of Southern Osseointegrated Fixtures [SOFs; Southern Implants (PTY) Ltd, Irene, South Africa (Southern)] in particular. In this study, we describe the success, long-term outcomes, complications, and overall impact and patient satisfaction in a series of patients who underwent treatment with SOFs in the craniofacial skeleton for the retention of auricular, nasal, and orbital prosthetics.

## METHODS

### IRB and Ethics Statement

This study was conducted in accordance with the Declaration of Helsinki. All patients gave their signed and informed consent to participate in the study. IRB review was sought. Because this was a retrospective review, ethics approval was not necessary, and a declaration confirming this has been submitted with this manuscript.

### Implant Characteristics

SOFs are self-tapping, cylindrical implants made of commercially pure Grade 4 titanium. The surface (bony part) of the implants is roughened by grit-blasting to enhance osseointegration. The fixtures are available in 3 lengths, namely 3.0 mm (IE3), 4.0 mm (IE4), and 6.0 mm (IE6).

### Study Design

This was a retrospective review of outcomes following use of SOFs for craniofacial reconstruction with an implant-retained silicone prosthesis. Patient data were collected from the Electronic Patient Record at our institution, and patient surveys were conducted between December 2024 and January 2025 at a hospital in the United Kingdom.

A total of 16 patients who underwent surgical implantation of SOFs between September 2013 and October 2024 were included. All patients were recruited from the clinic population managed by the senior author. Patients were included if they had accessible electronic records documenting their treatment journey. One patient was excluded from the implant outcome and complications analysis due to missing implant details. One patient had not completed treatment, and one had not yet received their prosthesis at the time of data collection. A further 4 patients were lost to follow-up and could not be contacted for the prosthesis satisfaction surveys. Therefore, only 10 patients were evaluated for their prosthesis usage and the satisfaction analysis (Supplemental Table 1, Supplemental Digital Content 1, http://links.lww.com/SCS/J702).

### Data Collection

We recorded information on patient demographics (age, gender, risk factors), surgical details, implant characteristics, ancillary procedures performed, the type and number of implants, their anatomic location, complications for each implant, and prosthesis type. Implant status at follow-up was also documented. PROMS were collected using a telephone survey, which included questions about prosthesis retention and use, delivery times, funding sources, satisfaction levels, and adherence to post-op protocols for care of the implants.

### Statistical Analysis

Descriptive statistics were used to summarize patient and treatment characteristics, complication rates, and PROMS. Patients with missing data for a specific variable were excluded from that analysis but included in others where relevant.

## RESULTS

### Patient and Treatment Characteristics

There were 9 men and 7 women, ranging in age from 15 to 78 years, with an average age of 44 and 4 months at the time of first surgery. The most common reason for treatment was trauma (7 patients, 43.75%), followed by microtia (5 patients, 31.25%) and oncological resection (3 patients, 18.75%). One patient underwent treatment after necrotizing fasciitis. The SOFs were used to fix an auricular prosthesis in 13 patients, an orbital prosthesis in 2, and both in 1 patient. Eleven patients had 3 SOFs each, 2 patients had 2 SOFs each, and 3 were implanted with a total of 6 SOFs, with 3 OI implants placed at each surgical site. In 3 cases, patients underwent insertion of 1 additional SOF each after their initial surgery (as a replacement) for a total of 58 implants inserted in 16 patients.

### Risk Factors and Ancillary Procedures

The most common risk factors were mild psychiatric disorders (7 patients, 43.75%) and the presence of soft-tissue scarring at the site of insertion (7 patients, 43.75%), followed by a history of smoking (6 patients, 37.5%). Other risk factors included pre-existing neurological dysfunction, compromised vascularity of the surgical bed, a history of craniofacial radiation therapy, poor condition of the craniofacial bone, and decreased bone healing potential due to an underlying systemic condition. Four patients underwent additional full-thickness skin grafting at the site of insertion, 4 patients underwent soft-tissue reduction, and 5 patients needed both skin grafts and reductions (Supplemental Table 2, Supplemental Digital Content 1, http://links.lww.com/SCS/J702).

### Implant Outcomes and Complications

Out of the 55 implants for which product codes were available, 28 were IE6, 20 were IE4, and 7 were IE3. The most common combination was 3 × IE6 (for 5 out of 15 patients). Of these, 10 implants failed to osseointegrate; 8 fell out, and 1 was removed at the patient’s request (due to non-use). In one case, an implant was surgically removed and another inserted into better quality bone (adjacent to the first implant), where it successfully osseointegrated. Importantly, in 6 of the 10 cases where the implants failed to osseointegrate, the implants had been placed into bone that was previously irradiated for treatment of a cancer. It is well recognized that radiation compromises the blood supply of the bone, and this is likely to have accounted for the failure of the implants to osseointegrate. In 1 of the 10 cases, loss of the implant was due to the presence of an (unexpected) cholesteatoma resulting in thinning of the cortex of the mastoid process to <1 mm (Supplemental Table 3, Supplemental Digital Content 1, http://links.lww.com/SCS/J702).

For SOFs that remained in place since implantation, survival ranged from 3 months to 11 years and 2 months (average 56 months). The most common complication was over-granulation, affecting 8 implants. Loosening of either the abutment or retention magnets was also common, affecting 5 implants. Infections of the adjacent soft tissues needing treatment occurred 6 times and affected 4 implants, including 2 adjacent implants that became infected twice at different times. Less common complications included pain (4 implants), hypertrophic scarring (3 implants, all in one patient), red peri-implant tissue (3 implants, all in one patient), clear yellow discharge (3 implants, all in one patient), wound dehiscence (2 implants), soft-tissue thickening (1 implant), and low-grade inflammation (1 implant) (Supplemental Table 4, Supplemental Digital Content 1, http://links.lww.com/SCS/J702).

### Prosthesis Usage and Satisfaction

Ten patients completed a prosthesis satisfaction survey covering a total of 37 implants (29 still in place at the time of the survey) and 12 prostheses (9 auricular and 3 orbital). Of these, 6 prosthetics were funded by the National Health Service (NHS), while the remaining 6 were funded privately. Three patients reported that they did not wear at least one of their prostheses; 2 because the OI implants were no longer in place to support them, and 1 due to the patient opting not to wear it. The remaining 8 patients (8 auricular and 1 orbital) described their prostheses as very stable when worn and wore them for more than 3 days a week (on average). Patient satisfaction and aesthetic outcome ratings were generally very high, with an average of 4.88 for patient satisfaction and 4.63 for aesthetic outcome (Likert scale of 1–5).

When we also included patients’ recollections for a prosthesis that was no longer in use, these ratings dropped to 4.82/5 for patient satisfaction and 4.5/5 for aesthetic outcome. These reductions were (mainly) due to the poorer outcomes for orbital prosthetics compared with auricular prosthetics. Of the 3 patients with an orbital prosthesis, only 2 provided ratings, reporting an average satisfaction of 4.5/5 and an aesthetic outcome of 3.5/5, compared with 4.88/5 and 4.75/5, respectively, for the 8 patients who were given an auricular prosthesis.

Most patients (6 out of 8) applied Savlon ointment [cetrimide (0.5% w/w) and chlorhexidine digluconate (0.1% w/w)] daily at the skin–implant interface of their OI implants. Six patients also reported using water and other cleaning methods, including pure alcohol and soap. One patient reported using Hydrex Pink (chlorhexidine gluconate 0.5% w/v in 70% v/v denatured ethanol) solution.

Prosthesis delivery times varied. Privately funded prosthetics were generally delivered within 6 months of implantation, with 1 patient receiving their prosthesis 3 months after the initial surgery and 2 patients receiving it after 6 months. Two patients were unable to recall the exact timeline due to the length of time that had passed since their procedure. For NHS-funded prosthetics, only 1 patient could recall the delivery time, which was 1 year after surgery. As previously mentioned, 1 patient was excluded from this analysis since they had not yet received their prosthesis. This patient underwent their second-stage surgery 4 years before this study, but has experienced significant postoperative complications, including a cholesteatoma and persistent granulation around their implants.

### Illustrative Cases

Two illustrative cases are presented to demonstrate typical clinical outcomes following implantation (Supplemental Table 5, Supplemental Digital Content 1, http://links.lww.com/SCS/J702).

Patient 01 sustained total avulsion of her scalp and right ear in an industrial incident. At the first stage, the soft tissues at the implant site were thinned so that the overlying skin was full-thickness over the periosteum. Three SOFs (1 × IE6 and 2 × IE4) were inserted into the temporal bone. Second-stage surgery to secure the abutments was performed 2 months later. At 2 months after the second stage, the patient experienced some over-granulation around the central abutment, which resolved with the topical application of Savlon cream. At 17 months after the second stage, the patient developed pain and discomfort associated with thickening of the tissues around the lower SOF implant. This was treated with local injections of triamcinolone and topical applications of Hydrex Pink (an alcohol-based antiseptic agent), with full resolution of symptoms. The implants remain stable at 2 years and 10 months, and the patient continues to wear her prosthetic ear (funded privately), outside of the house more than 3 days per week, and reported that her prosthesis is very stable. Both patient satisfaction and aesthetic outcome were rated as 4/5. Figure 1 illustrates clinical images showing the auricular region of patient 01 throughout reconstruction.

**FIGURE 1:**
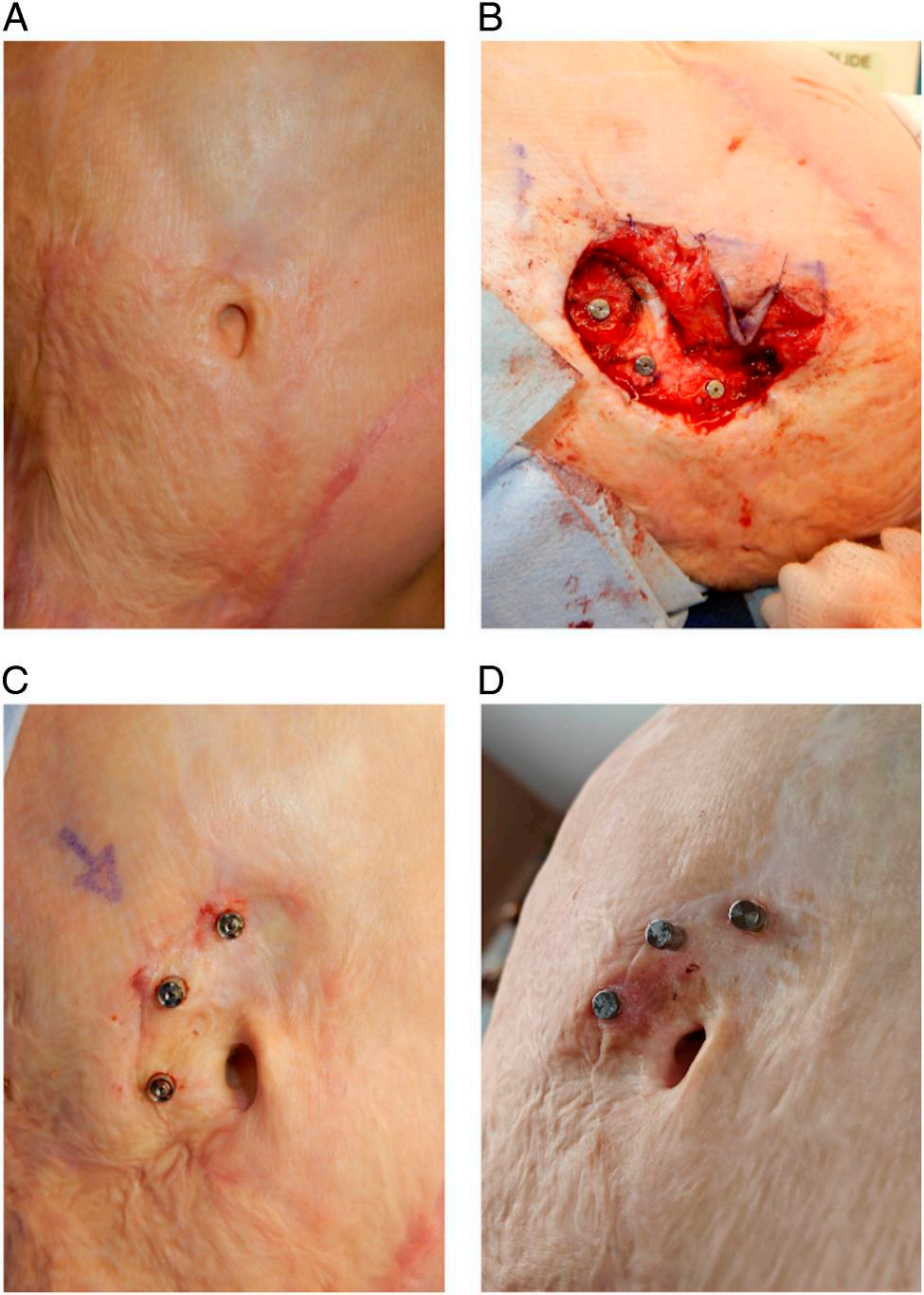
(A) Preoperative view of the patient’s ear. (B) Intraoperative view immediately following the first-stage surgery with SOFs in place. (C) Immediate postoperative appearance following abutment attachment in second-stage surgery. (D) Outcome at 17 months post–second surgery.

Patient 02 underwent reconstruction for a right microtia. At surgery, 1 × IE6 and 2 × IE4 SOFs were implanted into the right temporal bone. Second-stage surgery to secure the abutments was performed after 3 months. Following the first stage, the patient experienced a small seroma under the skin flap (4 and 10 days postoperatively), which was aspirated. At 3.5 years after surgery, they experienced one episode of low-grade inflammation observed around the middle implants (IE4). At 5 years and 8 months after surgery, all SOFs remain in place. A prosthesis was privately funded, and the patient wears it more than 3 days a week, reporting it to be very stable. The patient is currently on her third prosthesis, as she sees a prosthetist approximately every 2 years for replacement. One prosthesis required replacement earlier than expected after being damaged due to mask-wearing during the COVID-19 pandemic. Patient satisfaction and aesthetic outcome were both rated at 5/5. Figure 2 illustrates clinical images showing the auricular region of patient 02 throughout reconstruction.

**FIGURE 2:**
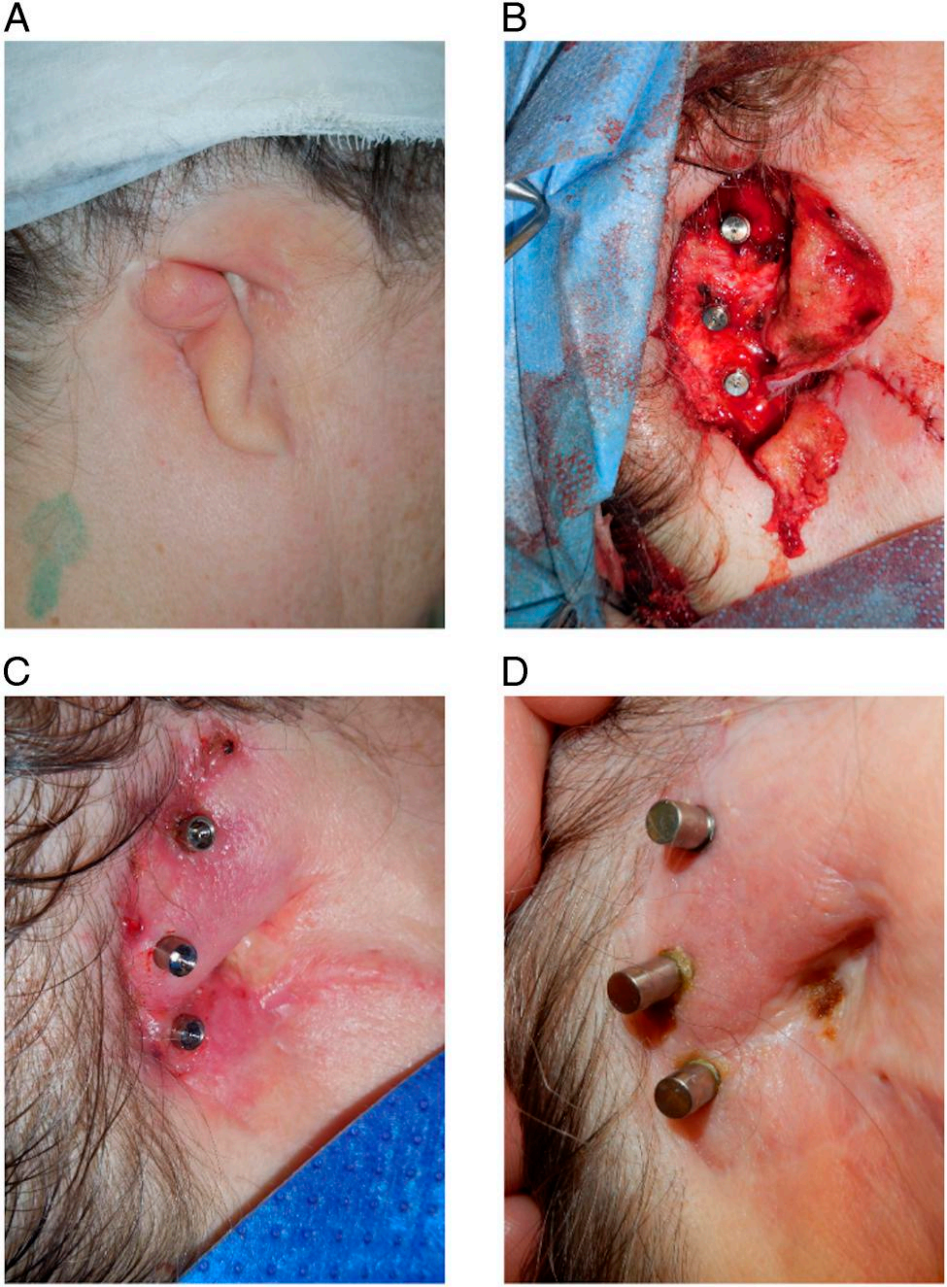
(A) Preoperative view showing right-sided microtia. (B) Intraoperative view immediately following the first-stage surgery with SOFs in place. (C) Immediate postoperative appearance following abutment attachment in second-stage surgery. (D) Outcome at 4 years and 10 months post–first surgery.

## DISCUSSION

Osseointegration of the SOFs succeeded in 48 out of 58 cases (82.76%). This concurs with previous studies using different OI implant systems, where reported survival ranged from 79.5% to 100%.^7,10,17,18,20–23^ For example, Sandelski et al^20^ reported a 86% survival rate at 5 years using Vistafix implants, which have a similar design to SOF implants. Figure 3 illustrates the minor differences in the design of SOFs and Vistafix implants. These images were taken from the respective manufacturers’ catalogs.

**FIGURE 3:**
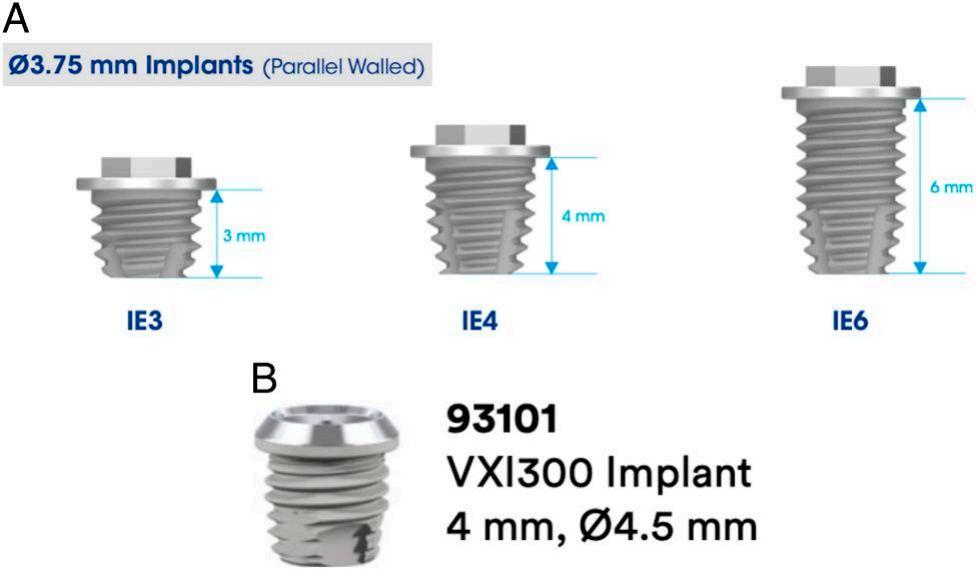
(A) Southern Osseointegrated Fixtures are available in 3 lengths. Image reproduced with permission from Southern Implants (Pty) Ltd, Irene, South Africa.^24^ (B) Vistafix 3 implant (4 mm length shown). © Cochlear Limited 2023. Image courtesy of Cochlear Limited.^25^

Notably, most of the SOF implants that failed to osseointegrate successfully (6 of 10) were placed into irradiated bone. ElKhasab et al,^26^ Alberga et al,^10^ and others,^17,23^ also reported on the increased risk of failure of OI implants placed into irradiated bone. Six of the 10 implants that failed to osseointegrate in our study were placed into the orbital cavity. Alberga et al^10^ found a marked decrease in the survival rate for orbital bone implants compared with auricular and nasal implants, especially after oncologic surgery. Therefore, whether our high failure rate was due to the poor quality of the bone at this particular anatomic location or to the fact that these patients also underwent radiotherapy to the bone (or both), it is clear that other surgeons have encountered similar difficulties. However, our small sample size prevented us from reaching any definitive conclusions.

In addition to loss of their implants, the patients in this study suffered from a similar list of complications to those encountered by others using different implant systems. The SOF adverse skin reaction rate that was classed as Holgers grade 2 was 3/55 (5.5%), affecting 1 patient, similar to multiple other studies' results of between 6.9% and 26%,^7,10,13,21–23,27^ and Holgers grade 3 for 8/55 (14.5%) implants, affecting 4/15 patients, which is similar to 1.5% to 15% reported in other studies.^7,10,13,21,22,27^ The complication rates for this study were at the upper limits of those seen in other studies. However, we speculated that this might have been because of the greater complexity of the type of patients treated in our unit.

Of the 10 patients who responded to our patient satisfaction survey, 8 (78%) were still using their prosthesis on a regular basis. This is similar to the rates described by Higgins et al^28^ underlining the value of this method of treatment for reconstruction of craniofacial defects. In addition, the average satisfaction score was 4.88/5, which compares favorably with other studies, which vary from ~2.16 to 4.69/5.^23,29–31^ Satisfaction with the appearance of their prosthesis was also high at 4.63/5 but differed depending on the anatomic site, with poorer outcomes for orbital compared with auricular prosthetics. However, given that we only had 2 patients with an orbital prosthesis (one of whom no longer wears their prosthesis), it was difficult to draw any firm conclusions on the reasons for this difference.

Although the sample size for this study was small, the observed implant survival and complication profile of SOFs are broadly consistent with previously reported outcomes for other (similar) implant systems,^7,10,13,17,18,20–23,27–31^ such as Vistafix and Branemark. However, we acknowledge several limitations of this study. The patient cohort was heterogeneous with respect to indication, prior treatment, and age. This heterogeneity reflects the range of clinical scenarios in which craniofacial prosthetic reconstruction may be performed, but it also limits the interpretation of outcomes in relation to underlying patient factors. In addition, the study interval was long (11 years), which contributed to several patients being lost to follow-up. This lowered the response rate to our telephone survey and increased the risk of recall bias when asking patients for details about the provision of their prosthesis after surgery. To be able to draw firm conclusions, we would need to gather more data on more cases. This is particularly true for patients undergoing orbital implantation.

Our study suggests that SOFs provide stable retention for craniofacial prostheses in the majority of cases, with generally manageable complications and high patient satisfaction. Larger prospective studies in more clearly defined patient cohorts are required to further evaluate these outcomes and adjust for patient-related factors.

## Supplementary Material
