# Supplementary material for: Clinical and Patient Satisfaction Outcomes from Southern Osseointegrated Fixtures for Craniofacial Reconstruction: A Retrospective Study
Source: J Craniofac Surg. 2026 Jun 18;37(10):2891–5. doi: 10.1097/SCS.0000000000013028 (PMC13645401; doi:10.1097/SCS.0000000000013028)
Supplement: Supplementary file 1 [file scs-37-02891-s001.docx]

***Supplemental Table 1: Patient Inclusion***

| **Category** | **Patients included** | **Implant Number** | **Exclusions and Reasons** |
| --- | --- | --- | --- |
| **Total patients** | 16 | 58 | --- |
| **Patient & treatment characteristics** | 16 | 58 | None |
| **Risk factors & ancillary procedures** | 16 | 58 | None |
| **Implant outcomes & complications** | 15 | 55 | 1 missing implant details |
| **Prosthesis usage & satisfaction** | 10 | 37 | 1 incomplete treatment 1 awaiting prosthesis 4 lost to follow-up |

***Supplemental Table 2: Risk factors & ancillary procedures***

| **Category** | **Number of Patients** |
| --- | --- |
| **Risk Factors** |  |
| Mild psychiatric disorders | 7 |
| Soft tissue scarring | 7 |
| History of smoking | 6 |
| Pre-existing neurological dysfunction | 3 |
| Compromised vascularity on the surgical bed | 2 |
| History of orofacial radiation therapy | 2 |
| Poor craniofacial bone condition | 1 |
| Decreased bone healing potential | 1 |
| **Ancillary Procedures** |  |
| Soft tissue grafting | 9 |
| Soft tissue reduction | 9 |

***Supplemental Table 3: Reasons for implant loosening***

| **Implant Type** | **Total** | **Failed to Osseointegrate** | **Potential Failure Causes** |
| --- | --- | --- | --- |
| **IE6** | 27 | 7 | Orofacial radiation (5 orbital) Poor condition of craniofacial bone (1 auricular) Other (1 auricular) |
| **IE4** | 20 | 2 | Orofacial radiation (1 orbital) Cholesteatoma (1 auricular) |
| **IE3** | 7 | 1 | Other (1 auricular) |

***Supplemental Table 4: Implant related complications***

| **Complication** | **Number & Type of Implant** | **Number of Patients** |
| --- | --- | --- |
| **Granulation** | 5 IE4 2 IE3 1 IE6 | 4 patients |
| **Infection** | 2 IE4 2 IE3 | 3 patients |
| **Loosening of abutment/superstructure** | 3 IE6 2 IE4 | 5 patients |
| **Pain** | 3 IE4 1 IE6 | 3 patients |
| **Hypertrophic scarring** | 2 IE6 1 IE4 | 1 patient |
| **Red peri-implant tissue** | 2 IE6 1 IE4 | 1 patient |
| **Discharge** | 2 IE6 1 IE4 | 1 patient |
| **Wound dehiscence** | 2 IE6 | 2 patients |
| **Soft tissue thickening** | 1 IE6 | 1 patient |
| **Low-grade inflammation** | 1 IE4 | 1 patient |

***Supplemental Table 5: Clinical Case Demographics***

| **Patient Number** | **Age at Surgery** | **Risk Factors** | **Reason for Treatment** | **Number and Type of SOFs** | **Prosthesis Type** | **Ancillary Procedures** |
| --- | --- | --- | --- | --- | --- | --- |
| **01** | 26 | History of smoking Soft tissue scarring | Scalp and ear avulsion due to industrial accident | 1 x IE6, 2 x IE4 | 1 x Auricular | Soft tissue reduction |
| **02** | 62 | None | Microtia | 1 x IE6, 2 x IE4 | 1 x Auricular | None |
